# Impact of ocean acidification on crystallographic vital effect of the coral skeleton

**DOI:** 10.1038/s41467-019-10833-6

**Published:** 2019-07-01

**Authors:** Ismael Coronado, Maoz Fine, Francesca R. Bosellini, Jarosław Stolarski

**Affiliations:** 1 Institute of Paleobiology, Twarda 51/55, PL-00-818 Warsaw, Poland; 2 The Mina and Everard Goodman Faculty of Life Sciences, Bar Ilan University, 5290002 Ramat Gan, Israel; 3 The Interuniversity Institute for Marine Sciences, P.O. Box 469, 88103 Eilat, Israel; 4 Dipartimento di Scienze Chimiche e Geologiche, Università di Modena e Reggio Emilia, Via Campi 103, 41125 Modena, Italy

**Keywords:** Electron microscopy, X-ray crystallography, Climate change, Ocean sciences, Biomaterials

## Abstract

Distinguishing between environmental and species-specific physiological signals, recorded in coral skeletons, is one of the fundamental challenges in their reliable use as (paleo)climate proxies. To date, characteristic biological bias in skeleton-recorded environmental signatures (vital effect) was shown in shifts in geochemical signatures. Herein, for the first time, we have assessed crystallographic parameters of bio-aragonite to study the response of the reef-building coral *Stylophora pistillata* to experimental seawater acidification (pH 8.2, 7.6 and 7.3). Skeletons formed under high *p*CO_2_ conditions show systematic crystallographic changes such as better constrained crystal orientation and anisotropic distortions of bio-aragonite lattice parameters due to increased amount of intracrystalline organic matrix and water content. These variations in crystallographic features that seem to reflect physiological adjustments of biomineralizing organisms to environmental change, are herein called crystallographic vital effect (CVE). CVE may register those changes in the biomineralization process that may not yet be perceived at the macromorphological skeletal level.

## Abstract (web summary)

Coral fossils can record climatic history, but teasing apart environmental signals remains a challenge. Here the authors show that crystallographic changes in coral skeletons grown under high CO2 conditions could be used as a sensitive pH proxy, enabling measurement of ocean acidification back in time.

## Introduction

The past twenty years have borne witness to remarkable advancements in the understanding of scleractinian coral biomineralization. A paradigm of a purely physicochemical model of skeleton formation was replaced by a model of biologically controlled mineralisation (biomineralization). Consequently, the skeleton is no longer considered to be composed of’single orthorhombic crystal(s) of aragonite’^1^ but as a composite structure of hierarchically assembled mineral and organic phases, with the organic phases typically referred to as the organic matrix, OM (organic macromolecules consisting of proteins, carbohydrates, and lipids). In contrast to inorganically precipitated aragonite, coral bio-aragonite shows several distinct crystallo-chemical properties such as crystal morphology (from nanogranulae to microfibers^2–5^), controlled textures (microstructural patterns related to phylogenetic position of coral^3,6,7^), preferential crystallographic orientation^5,8–10^, and heterogenous biogeochemical composition^11–13^. These features of the coral biominerals are the outcome of highly variable physiological activity of epithelial cells (i.e., calicoblastic ectoderm) that control: secretion of organic phases into calcification medium; ion transport from external environment; formation of an amorphous phase, a direct substrate for crystallisation via amorphous particle attachment mechanism^14–16^.

Physiological, species-specific control over mineralisation modifies some biogeochemical characteristics of coral skeleton, overriding the environmental signatures expected from thermodynamic equilibrium precipitation, which is called vital effect^17,18^. For example, rapid accretion deposits (RAD, central microcrystalline areas of a septum, traditionally called centres of calcification), in contrast to thickening deposits (TD, bundles of fibres formed outside the RAD), are enriched in several elements such as Mg, Sr, S, B, Ba and N^12,13^ and organic phases^3,13^, whereas several isotope ratios, such as δ^13^C, δ^18^O and δ^11^B, are depleted in RAD. Purely physicochemical models of biomineralization, such as Rayleigh fractionation, are not capable of explaining these fine-scale trace element variations^19^. Contrary to physicochemical models, changes of environmental factors like high *p*CO_2_ (ocean acidification), temperature, and chemical composition of seawater affect primarily physiology and biochemical cycles of biomineralizing organisms, and only indirectly the process of biologically controlled CaCO_3_ formation^20^.

The effects of ocean acidification (OA) on coral biomineralization have been identified in natural (Great Barrier Reef in Australia^21^) and experimental conditions emulating acidification scenarios predicted by the end of the 21st century (pH drop *ca*. 0.3–0.5 units)^22^. These studies underline distinct decreases of calcification rate, from 13% in natural waters to 30–60% in mesocosm experiments;^23,24^ occurrence of deformations in juvenile stages and during coral larval attachment;^25^ thinning of skeletons and increasing of porosity;^26,27^ dissolution;^28^ and tissue-specific apoptosis in adult colonies that may result in loss of coral coloniality^29^. Although some corals are capable to adapt to changes^30,31^, the combination of acidification and high temperatures typically have a detrimental effect on coral survival^23,32^. Experiments with common reef-building coral *Stylophora pistillata* suggest diverse physiological and morphological responses of this coral to high *p*CO_2_ conditions^26,30,33^.

Building upon these studies we have investigated unexplored to date effects of OA on crystallographic features, the lowest and most fundamental level of skeleton organisation. In the experiment, genetically identical (clones) colonies of *S. pistillata* were cultured at different pH treatments (8.2, 7.6 and 7.3) and at ~25 °C (Table 1) for a 14 months acclimation period. Next, the cleaned skeletons were characterised by a suite of microscopic, crystallographic, and thermal analysis techniques. We found that the bundles of fibres are more rounded at pH 8.2 than in acidified conditions (at pH 7.3 and 7.6) and the crystal orientation is better constrained at higher *p*CO_2_ conditions. On the other hand we found some nonlinear anisotropic distortions of aragonite lattice parameters in crystallite size and the microstrain. These modifications result from the increased amount of intracrystalline organic matrix and changes in water content, at higher pH (i.e., 7.3 pH). These fine-scale skeletal responses to acidified conditions suggest the complexity of physiological machinery that regulates coral biomineralization. They suggest that crystallographic features can be sensitive proxies of these physiological processes, which herein are called crystallographic vital effects (CVE).

**Table 1:** Carbonate chemistry parameters of treatments and control

pH NBS	TA (μeqv kg^−1^)	DIC (μmol kg^−1^)	pCO_2_ (μatm)	CO_2_(aq) (μmol kg^−1^)	HCO^3−^ (μmol kg^−1^)	CO_3_^2−^ (μmol kg^−1^)	Ωarg
8.2	2501	2122	387	10.6	1846	265	4.02
7.6	2499	2431	1917	52	2295	82	1.25
7.3	2501	2544	3898	107.1	2393	44	0.67

*Note*: Parameters calculated from pH, total alkalinity (TA), temperature (25 °C), and salinity (40 ppm) using the programme CO_2_SYS

## Results

### Structural features

**Fig. 1:**
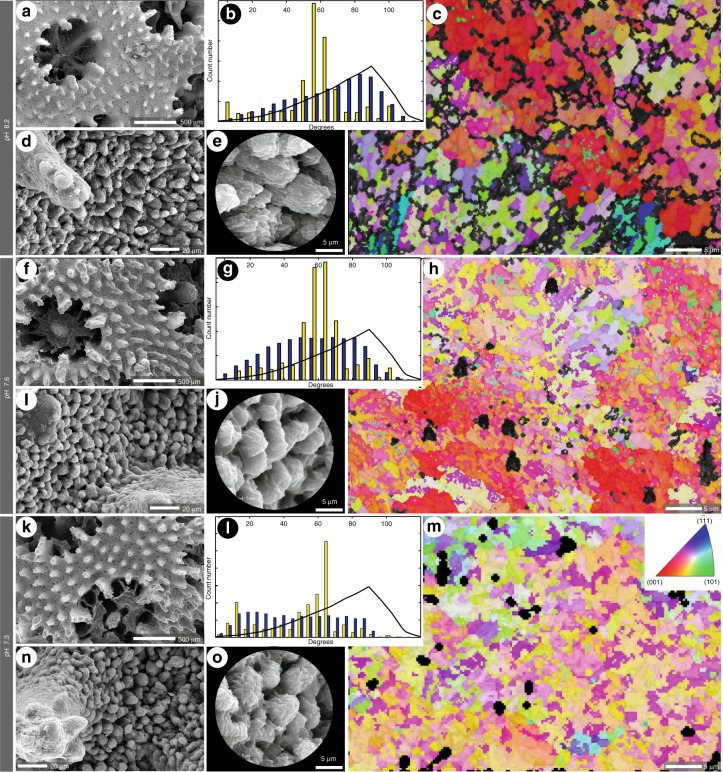
Structural features of *Stylophora pistillata* cultured at pH conditions. Overall view of the colony surface with corallites and spiny coenosteum (**a**, **f**, **k**). Enlargements of the coenosteum showing granulated surface composed by bundles of fibres (**d**, **i**, **n**; details of the texture of bundles in zoomed areas (**e**, **j**, **o**). Note that the texture of bundles grown in 7.3 pH and 7.6 is more rounded than those grown in pH 8.2. EBSD orientation maps showing the crystallographic arrangement of thickening deposits crystals (**c**, **h**, **m**). Colour-coding assigns a colour to each possible orientation. Histograms (**b**, **g**, **l**) representing correlated (yellow bars) and uncorrelated (blue bars) misorientation of crystals (black line represents random distribution computed for this crystal symmetry). Correlated misorientation describes fibre-to-fibre relationships (in contact), whereas uncorrelated misorientation described relationships between fibres without direct contact (e.g., between different bundles). Misorientation is measured regardless of coral sectioning (morphological orientation of aragonite fibre bundles)

Colonies cultured at all pH treatments show, at low magnification, a surface with spiny coenosteum and corallites with six primary septa, occasionally six shorter secondary septa (Fig. 1a, f, k). Morphological differences between coralla from various pH treatments are not very distinct but we noted that size of corallite calyxes increased with decreasing pH (consistently with Tambutté et al.^26^), and the samples from reduced pH conditions show *ca*. 2.2 (pH 7.6) and 1.6 (pH 7.3) times longer spines in comparison to samples from pH 8.2. Enlargements of the coenosteum show granulated surfaces composed of bundles of fibres (Fig. 1d–e, i–j, n–o) of thickening deposits (TD) and smoother but polycentric rapid accretion deposits (RAD, Fig. 1d,i,n). In acidified conditions (at pH 7.3 and 7.6) the texture of bundles of fibres is more rounded than at pH 8.2 (Fig. 1e, j, o). After etching of the polished sections, the bundles of fibres at pH 7.6 appear more rounded and composed of shorten fibres (Supplementary Fig. 1c, d), whereas at pH 7.3 the bundles seem more irregular (Supplementary Fig. 1e, f), similar to pH 8.2, but the crystals have an appearance of coarse granular aggregates, in contrast with the flat and slightly curved needles of pH 8.2 (Supplementary Fig. 1a, b). Noteworthy, the organic remains connecting fibres and surrounding them are more visible after etching in those samples cultured at lower pH (7.6 and 7.3).

### Crystallographic features

Crystallographic characterisation of *Stylophora* skeletons have been addressed using Electron Back-Scatter Diffraction (EBSD), which is an electron-beam technique that allows to identify mineral phases, measure the crystallographic orientation of crystalline elements and evaluate the spatial and crystallographic relationships between crystals (e.g., misorientation, more details below). Two-three equivalent areas (i.e., mid branch of colonies) of different *Stylophora* colonies (*ca*. 360 × 300 µm) per pH were analysed (two colonies per pH and one natural sample). The other used technique, X-ray powder diffraction (XRPD) is a non-destructive, routinely used technique in phase analysis, the calculation of crystallographic unit cell, determination of crystal structure, and the estimation of crystallite size and the macrostrain and microstrain of the crystal lattice. Two individual colonies of *Stylophora* per pH were analysed with the purpose to identify the present mineralogy, calculate the lattice parameters and microstructural features (macrostrain and microstrain and crystallite size). EBSD and XRPD analyses confirm that skeletons of *S. pistillata* at all pH treatments are composed of aragonite. The microstructure of samples is very well observed in band contrast images (BC) from EBSD analysis because the edges of bundles of fibres have low diffraction intensity (Supplementary Fig. 2a–f). Other areas, as RAD, have likewise low diffraction intensity with few aragonite points indexed (Supplementary Fig. 2e), due to their amorphous structure (a possible combination of protoaragonite and ACC^5,14,15^). The diffraction quality was compared by examining the grey values of the BC images (Supplementary Table 1) following the methodology proposed by Fitzer et al.^34^ in TD regions (two samples each pH). This method allows assessing how crystalline is a mineral structure according to diffraction quality (development and quality of kikuchi patterns) and subsequently transferred to a colour scale. Light greys indicate crystalline materials whilst dark greys, even black, point to less or even noncrystalline materials. Amorphous or not well crystalline materials, as protoaragonite (pAra), do not produce kikuchi patterns or with enough quality to be detected by EBSD and for instance are coloured in dark greys (Supplementary Fig. 2). Fitzer et al.^34^ assessed diffraction correlating with ACC presence in molluscs. At acidified conditions the diffraction intensity is higher, being the highest at pH 7.6, and it is evident the small variability between samples and selected areas (Supplementary Fig 2g–i), although the mean of three groups are significantly different (*p* < 0.005) unpaired Student’s *t*-test. Crystallographic orientation images (COI) show a coherent crystallographic arrangement (Fig. 1c, h, m) between all samples (two-three samples at each pH), with *c*-axis, contained in the plane (001), parallel to morphological axis of crystal fibres (Supplementary Fig. 3). High crystallographic changes are more evident between bundles than within the bundles (Fig. 1c, h m). The COI (Fig. 1) and pole figures (Supplementary Fig. 3) of selected planes (100), (010), (001) and (222) display the highest crystallographic control of the bundles at low pH (Supplementary Fig. 3), namely, the dispersion of orientations is lower, while at higher pH samples the orientations are rather randomly distributed. At pH 8.2 the maximum pole in (001) is widespread, whereas it is well constrained at pH 7.3 (Supplementary Fig. 3). Even when a turbostratic distribution^35^ is observed at the plane (222), i.e., when *a* and *b*-axes rotate around the *c*-axis (Supplementary Fig. 3), the dispersion in terms of inclination of crystallographic axes is lower, less dispersed, at low pH (planes (100) and (010) of Supplementary Fig. 3).

**Fig. 2:**
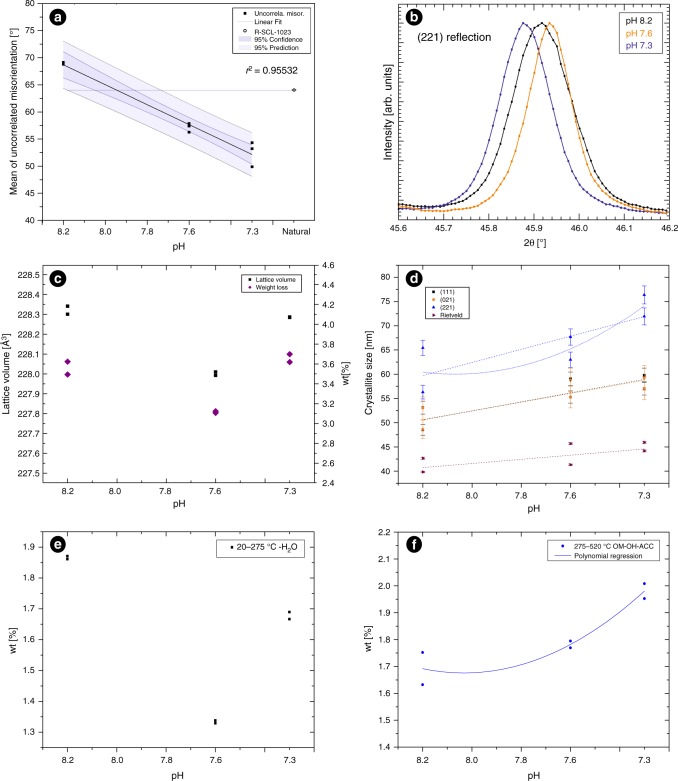
Crystallographic changes in the skeleton of *Stylophora pistillata* cultured at different pH conditions. **a** Mean of uncorrelated misorientation versus pH and an external natural sample (R-SCL-1023) as a control point. The black line is the linear regression of samples cultured at different pH conditions. The decreasing response to acidification in the mean of uncorrelated misorientation indicates that the crystallographic control exerted by coral at microscale increases at low pH. **b** Comparison between angular positions of a selected diffraction peak taken, belonging to (221) reflection. Note the shift in the angular position between pH 8.2 on regard to 7.6 samples to higher angles and 7.3 to lower angles. **c** Lattice volume calculated for each pH (black squares) and the total weight % loss (purple diamonds) in the range 20–520 °C. **d** Calculations of crystallite size for the skeletons of the 3 different pH treatments. Note the increment of all of them, independently of the method. The error bars represent calculated error of crystal size after refinement of UVW-parameters. Plane (221) is showing a larger crystallite size. Curved blue line corresponds with polynomial regression, whereas straight lines correspond with linear regression. **e** Water regime (20–275 °C). **f** OM-OH-ACC regime (275–520 °C). Blue line correspond with the polynomial regression of the points (*r*^2^ = 0.843). OM organic matrix, OH OH groups, ACC possible Amorphous Calcium Carbonate

Another compared crystallographic feature is the misorientation, which is a difference between two orientations e.g., of two crystals, which can be in direct contact by a grain boundary (correlated misorientation) or not (uncorrelated misorientation). The misorientation was analysed for the TD areas in all the samples (2–3 each pH). The correlated misorientation is uniform in all pH treatments (Fig. 1b, g, l, yellow bars), because the disorientation between crystals remains constant, regardless of pH. On the contrary, uncorrelated misorientation is changing with the pH, from almost random orientation at pH 8.2 (Fig. 1b, blue bars), to lower angle values of disorientation at pH 7.3 (Fig. 1l), *ca*. 28% less from 69° to 50° of mean. This parameter is directly related to the disorientation between bundles. The mean of uncorrelated misorientation is showing a linear correlation with the pH, at acidified conditions the skeletons are better organised (Fig. 2a), although the variability is increasing at low pH (higher at pH 7.3). The mean of uncorrelated misorientation of a natural colony of *S. pistillata* collected in the Gulf of Eilat (Israel) was plotted together with the experimental data, with the purpose to calculate the pH recorded by the skeleton (Fig. 2a). The calculated value is from pH 8.05 to 7.87 in the interval of 95% of confidence and it is consistent with the mean of pH for this region (*ca*. pH 8.1)^33,36^.

The incorporation of some trace elements (such as Mg) and intracrystalline organic matrix in the CaCO_3_ structure causes shifts in the reflections of XRPD patterns and subsequent lattice distortions ^37–40^ (i.e., macrostrain). The XRPD pattern of samples from all treatments (2 individual colonies each pH) were refined using the Rietveld method and the lattice parameters (Supplementary Table 2), and microstructural data were calculated (i.e., crystallite size and microstrain, Supplementary Table 3). The distortions in the lattice parameters of analysed samples do not show a linear correlation (Fig. 2b, c), although low pH samples show negative distortions (shrinkage) in all axes and a consequent lattice volume reduction in comparison with pH 8.2 (Supplementary Table 2). The macrostrain (Supplementary Table 2) is slightly higher at pH 7.6 samples (Δa/a(%) = −5.23E−02; Δb/b(%) = −5.11E−02; Δc/c(%) = −4.93E−02) than at pH 7.3 (Δa/a(%) = −7.2E−03; Δb/b(%) = −8.1E−03; Δc/c(%) = −8.2E−03), with pH 7.3 being close to pH 8.2. In spite of the small distortions of lattice parameters in each sample, pH 7.6 samples show a shift to higher values of 2θ and samples of pH 7.3 to lower values, taking 8.2 as reference (Fig. 2b). Crystallite size and microstrain were calculated from peak broadening of the full spectra and individual XRPD reflections (more details in Experimental section) for the planes (111), (021) and (221). Regardless of the method (Caglioti or Scherrer), the samples show an increase of crystallite size at pH 7.3 and 7.6 (Fig. 2d, from 48 to 60 nm for (111) reflection) and a subsequent microstrain decrease (Fig. S4, 0.119% to 0.098% for the (111) reflection). Although the crystallite size varies in each method, the values calculated for pH 8.2 sample are consistent with the nanocrystal size observed in *S. pistillata* by AFM^4,41^. The variation in crystallite size is the same for the planes (111) and (021) and they have a comparable slope to Rietveld calculations (Fig. 2d), while the plane (221) the crystallite size varies largely (Fig. 2d).

### Water and organic matrix estimation

Weight and energy changes can be identified during the thermal treatment of chemical substances (by the study of endothermic and exothermic reactions that produce). Weight loss caused, for example, by the release of volatile compounds (e.g., dehydration processes or losses in CO_2_, OH, SO_3_) or structural decomposition or transformation of material can be determined by the use of a thermobalance. Thermogravimetric analyses of heterogeneous compounds provide characteristic features for their identification (as structural water or organic content) and therefore it is very useful in the analysis of complex materials (such as polymers or composites materials like biominerals). Weight losses were calculated by TG-DSC analysis for all pH treatment skeletons (two samples for each pH) with the purpose to evaluate the contribution and variation of different structural components as water (in all their forms) and OM (Supplementary Fig. 5). The selected range of study is between room temperature *ca*. 20 °C to 520 °C, where the water and the main organic components are decomposed^42^ and the monotropic transformation of aragonite to calcite, which occurs at ~418 °C^43^. Some organic remnants could persist at higher temperatures (even ~800 °C^44^), even when the total CaCO_3_ decomposition to CaO has started at ~600 °C^45^, but we assume here that these are insignificant. The derivative weight curve (from 150–520 °C) shows three exothermal bands with irregular morphologies identified in all analysed samples, which correspond with different slopes in the weight loss curve (Supplementary Fig. 5). The derivative weight curve was deconvoluted getting several contribution bands, seven temperature intervals were finally selected (Supplementary Table 4, Fig. 5) and the weight losses were measured in accordance with changes in the slope of each selected region (Supplementary Table 4). The total weight losses of the three pH samples, between 3.1 and 3.7 wt% (Supplementary Table 4, quite consistent with the calculated by Reggi et al.^46^), have not a lineal response to acidification. The samples from pH 7.6 lost less weight than 8.2 and 7.3 samples, being these last, higher than 8.2 (Fig. 2). Two main weight loss regimes could be separated:^42,46^ derived from water (0–275 °C, including fluid and rigid water molecules based on ACC^47^, which could be trapped in site defects of the structure) and other thermal region derived from OM + ACC + OH_groups_ (275–520 °C, including organic matrix s.l^42^., trapped OH molecules released by dehydration of OM during thermal treatment^42^ and the hydrated forms of ACC (*sensu* Mass et al.^15^). Radha et al.^48^ established that the temperature of crystallisation of ACC to another more stable phase (calcite or aragonite) in both synthetic and biogenic ACC is between 313–329 °C. After deconvolution of derivative weight curve of *Stylophora pistillata* samples, a strong contribution was observed at *ca*. 316 °C (zone 4b, Supplementary Fig. 5, Table 5) of the exothermal peak *ca*. 300 °C, causing a strong shoulder at the peak in all the samples analysed. This strong contribution has been recognised as potential ACC.

The two thermal regimes identified have different responses to acidification: thermal region of water (150–275 °C) has a nonlinear response to pH variation and it is similar to the total weight loss, except for the samples cultured at pH 7.3, where the weight losses are lower than pH 8.2 samples (Fig. 2e, f). On the other hand, the OM + ACC + OH_groups_ (275–520 °C) thermal region has a lineal response to pH (Fig., 2e, f), increasing the weight loss at acidified conditions (from 1.63 to 2.00 wt%).

## Discussion

Previous studies on effects of ocean acidification in *Stylophora pistillata* have shown that higher *p*CO_2_, reduced pH in seawater, induces changes in the physiology (such as tissue biomass, chlorophyll changes, zooxanthellae cell density^26,30^); the morphology (such as calcification rate, calicular diameter, porosity, skeletal density^26^) and the skeleton biogeochemistry (such as stable and boron isotopes and proteins content^26,27,30^) and with diverse responses (linear or nonlinear). To date, however, it remained unknown how these changes could affect the fine-scale organisation of the skeleton. Our findings highlight that some the crystallographic properties of analysed *Stylophora pistillata* skeletons could be forced by anatomical and physiological changes in the coral organism in response to seawater acidification.

Tambutté et al.^49^ provided evidence that the skeleton is a fingerprint of the soft tissues arrangement. The calicoblastic ectoderm provides confined space for biomineralization and directly controls the crystal growth by organic template formation, the ions input by active transport from extracellular environment and regulates the shape of bundles of fibres. Different fibres bundle morphologies in *S. pistillata* were described and directly associated with different morphology of calicoblastic cells. Tambutté et al.^26^ showed that diameter of skeleton corallites increase linearly with acidification (increasing of porosity), whereas the thickness of structures, such as septa, decrease (decreasing skeletal density). This phenotypic strategy, in which the skeleton thickness is reduced, could explain the better constrained crystallographic orientation at low pH (linear depletion of uncorrelated misorientation, up to *ca.* 28% less) in TD areas by a calicoblastic ectoderm alignment. Modifications in the tissue arrangement may affect the crystallographic organisation between the fibre bundles (uncorrelated misorientation), whereas the crystallographic organisation within bundles of fibres (correlated misorientation) is kept constant along the experiment. It should be highlighted that at high pH the uncorrelated misorientation (i.e., 8.2) is confined under random distribution (Fig. 1b) for the pertinent theoretical symmetry and space group (orthorhombic, *Pmcn*) and pole figure shows an irregular pole maximum in the plane (001). Whereas at low pH (7.3) the uncorrelated misorientation pattern is out of theoretical random distribution towards low angles (more co-oriented crystals) and pole figures show a pole maxima more constrained. The linear constraining of the crystal orientation along the pH gradient, as a consequence of morphological changes produced in the skeleton, is a surprising finding opposite to the less constrained crystallographic orientation observed in some other calcifying organisms such as molluscs (i.e., *Mytilus* shells) with the increasing of pCO_2_^34,50^.

Microstructural changes identified here for first time in *S. pistillata*, seem to reflect changes in pH (e.g., roundness of fibre bundles and the texture of individual needles change) but the effects are nonlinear (fibres bundles of pH 7.6 skeletons are shortest, and fibres of pH 7.3 skeletons show coarse and granular texture). Considering that the pH is not significantly decreased in the calcification medium and the corals maintain the Ω_arg_ at elevated levels^27,51^, changes in the morphology of fibres and fibres bundles should be rather related with changes in the crystallisation conditions other than the pH, inasmuch as physico-chemical factors were kept constant during the experiment. Accordingly, the changes produced by high seawater *p*CO_2_ could directly affect other parts of the system, such as coral and/or symbiont physiology and affect microstructural and crystallographic levels of hierarchical organisation of the skeleton.

At higher crystallographic resolution, differences in lattice parameters were detected in samples from high seawater *p*CO_2_. These changes are closely related to the water and organics content estimated by TG-DSC analyses. Lattice volume and macrostrain variations are correlated with the total weight loss changes due to structural water + OM + ACC trapped into the crystals and corroborated by the angular

XRPD shifts (Fig. 2b), which are similar to those observed in other biogenic aragonite (i.e., molluscs) on regard to geological aragonite^38^. The samples cultured at pH 7.3 have similar lattice volume and weight losses to pH 8.2 samples (a short difference of ΔV/V = −0.002 to −0.007% and wt (%) = −0.003 to +0.2). On the other hand, discernible changes are found at pH 7.6 where the lattice volume is reduced *ca.* 0.15% and the weight loss *ca.* 0.5%. A similar response to OA between the thermal water region and the lattice volume distortions is observed (0–275 °C, Fig. 2c, e, f, Supplementary Table 2), suggesting that the water content is the main source of lattice distortions. Nonetheless, the weight loss for pH 7.3 at the thermal water range is lower than in pH 8.2 samples, indicating the organics contribution to the total weight loss is higher for samples cultured at that pH (OM + ACC + OH_groups_, 275–520 °C, Fig. 2e, f). The organic content (OM + ACC + OH_groups_) increases with the seawater *p*CO_2_, co-varying with a significant rise in crystallite size, mainly affecting the plane (221), and a small decrease of microstrain (Fig. 2d–f and Supplementary Fig. 4). This boosting of OM content and the subsequent rise of crystallite size could explain the textural changes observed at microscale at pH 7.3 samples, in which the intercrystalline OM remains are more visible around the fibres bundles (Supplementary Fig. 1f). The increased organic matrix content was observed during OA by the protein estimation of the coral tissue^30^ and the skeleton^26^. Both experiments support the idea that the pH produces a thickening of coral tissue and massive production of organics^30^, which is transferred to the skeleton during the biomineralization^26^. The role of these organics at low pH biomineralization conditions is unclear, but Tambutté et al.^26^ suggested that it may promote calcification under less favourable Ω_arag_. Raybaud et al.^51^ observed that, despite the decreasing of Ω_arag_ of seawater, the *S. pistillata* has the ability to buffer the solution and control the chemistry (increasing of total alkalinity) of calcification medium. Accordingly, herein observed weight loss in the organic thermal region is consistent with the increased formation of the organic matrix at lower pH, which could have the capacity of buffering pH changes as proposed by Raybaud et al.^51^. Consequently, Ω_ara_ changes in calcification medium by acidification would vary the coral calcification rates.

Coral skeletons belong to the most widely studied palaeoclimate and palaeoenvironmental archives, however, in contrast to pure calcium carbonate precipitates, show several vital effects that hinder straightforward physico-chemical interpretations. A better understanding of factors that are responsible for those vital effects is a key to the accurate assessment of present-day and the past effects of increased seawater *p*CO_2_ (ocean acidification). Our findings highlight that physiological changes in *Stylophora pistillata* in response to long-term experimental ocean acidification (OA), do not dramatically alter skeletal macromorphology (reported resilience^33,52^ to OA) but affect its fine-scale properties. These crystallo-chemical responses (linear and nonlinear) suggest the complexity of physiological machinery that regulates coral biomineralization and points to highly sensitive proxies of these processes, recorded at the lowest level of the coral skeleton, herein called crystallographic vital effect (CVE). Nonetheless, the reproducibility of the CVE should be explored in other coral species in future work. Although CVE is subtle in examined *S*. *pistillata* samples, some of them i.e., the variation of uncorrelated misorientation of crystals, show a linear correlation with the seawater pH (Fig. 2a). These parameters are not prone to change due to decomposition of intraskeletal organic matter during diagenesis^53^, thus may become a new crystallographic tool for detecting OA in well preserved aragonitic fossil corals supporting other well established geochemical tools e.g., boron isotopes^54^. In the long run (colony life span) crystallo-chemical changes may scale up at higher levels of skeletal hierarchy and potentially affect biomechanical properties of the reef structure^41^.

## Methods

### Coral culturing conditions

Small colonies of *Stylophora pistillata* corals were cultured in the experimental system at the Interuniversity Institute for Marine Sciences in Eilat (IUI) (29°30′N 34°55′E). The seawater for the system was pumped from a depth of 30 m at the IUI reef into 1000 L tanks, where the pH was regulated (see Table 1 for the seawater chemistry). Colonies were dyed with Alizarin Red (Sigma–Aldrich, USA) in order to label the beginning of the experiment in the skeleton after that were cultured over 14 months at different pH conditions. The pH values (i.e., 8.2, 7.6 and 7.3) were achieved by bubbling pure CO_2_ gas stored in a cylinder through seawater to reach the desired pH. A pH electrode (S-200C; Sensorex, California) was located in each tank and connected to a pH controller (Aquastar; IKS ComputerSystem GmbH, Karlsbad, Germany) in order to control the gas flow. A pH deviation within the tank triggered the computer to activate a solenoid to either increase or decrease the flow of CO_2_, as necessary. The pH data were recorded using monitoring software (Timo, Matuta, Germany). The pH in the aquarium was monitored and logged every two minutes and the electrodes were calibrated once a week, or when discrepancies were detected. The seawater temperature was maintained at ~25 °C, using a combination of an array of 150 W BluClima aquarium heaters (Ferplast Spa, Vicenza, Italy) and an air-conditioner in the wet-laboratory. Water motion in the tanks was maintained by power heads (Atman, At-301, China). Carbonate chemistry parameters of treatments and control were calculated from pH, total alkalinity (TA), temperature (25 °C), and salinity (40 ppm) using the programme CO2SYS^55^.

### Sample preparation

Dry colonies of *Stylophora pistillata* cultured at different pH were prepared by removing the soft tissue using a water jet and the persistent remains by overnight immersion in 4% sodium hypochlorite (NaOCl) solution, rinsed with distilled water and air dried at room temperature. The colonies were cut with a diamond saw, using as landmark an Alizarine Red stain (details in Krief et al.^30^) and divided for different preparations: (1) samples were embedded using epoxy resin and cut in a transverse section, latterly thin-sections (*ca*. 30 μm thick) were prepared and other sets of samples were cut forming a slide of 1 mm thickness (thick slides); (2) intact pieces of coral skeleton were gently grinded using an agate mortar reaching a powder fraction lower than 5 μm.

### Skeletal microstructure

Skeletal microstructural features were studied and photographed using a transmitted light microscope Nikon Eclipse 80i and a scanning electron microscope (SEM) model Philips XL 20 SEM, located at Institute of Paleobiology of Polish Academy of Sciences (Warsaw, Poland). Specimens were observed in intact colonies and in polished and etched samples (by *ca*. 20 s etching in 0.1% formic acid solution). The etched samples were rinsed with distilled water and air dried at room temperature. Once dried, the samples were mounted on stubs with double-sided adhesive tape and sputter coated with a conductive platinum film.

### Electron backscatter diffraction (EBSD)

The surface sample (of thick slides) was polished with alumina of 1 µm, 0.3 µm and 0.05 µm and finally polished with colloidal silica (0.05 µm). Before analysis, samples were coated with a thin layer (*ca*. 2 nm) of carbon using a high vacuum coater Leica EM ACE600. The EBSD study has was carried out with an Oxford Nordlys camera mounted on a Field Emission Scanning Electron Microscope (FE-SEM) JEOL JSM 6500 F located in the Electron Microscopy Laboratory of the Spanish National Research Centre for Metallurgy (CENIM-CSIC). EBSD data were collected with CHANNEL 5 software at high vacuum, 10 kV, large probe current and 15 mm of working distance. EBSD patterns were collected at a resolution of 0.1–0.2 µm step size for crystallographic maps using the unit cell settings characteristic of aragonite: ‘Pmcn’ symmetry and *a* = 4.96509 (2) Å, *b* = 7.97226 (3) Å, *c* = 5.75004 (2) Å (Stolarski et al.^39^) estimated for *Favia* coral using X-ray powder diffraction with synchrotron radiation. Two-three areas of different colonies (*ca*. 360 × 300 µm) per pH were analysed (two colonies per pH and one natural sample). EBSD data were processed using CHANNEL 5 from Oxford Instruments. In this study, EBSD data are represented by crystallographic maps, band contrast images, pole figures and plot the ODF (orientation density functions), which represent the stereographic projection of crystallographic planes in reference to the (100), (010), (001) and (222) aragonite planes. MATLAB^TM^ toolbox MTEX^56^ was used to calculate the correlated and uncorrelated misorientation. The misorientation calculus included all orientations detected during the EBSD analysis. In the case of uncorrelated misorientation, the algorithm compared one orientation with all those in not direct contact with it.

### X-ray powder diffraction (XRPD)

The X-ray powder diffraction measurements were carried out at the CAI of X-ray diffraction in the Complutense Unversity of Madrid (Spain), using a PANalytical diffractometer, model X’Pert PRO Alpha 1 X-ray. The working conditions were: Cu Kα (1.540598 Å), 45 kV and 40 mA, using a Ge monochromator for the (111) diffraction reflection. The diffraction patterns were registered by reflection mode while rotating the sample around Φ angle in a continuous scan mode from the specimens at room temperature. The step-length was 0.0084° and a continuous time of 400 s/step, acquiring 8975 points and the 2θ (°) range 15–90° was covered over a period of 8 h. Two individual colonies per pH were analysed.

The structural parameters were refined by Rietveld analysis using the Fullprof software and a Pseudo-Voigt peak shape function^57^. The refined model was based on 16 structural parameters and 12 instrumental and background parameters. Lattice distortions, crystallite size and microstrain analyses were performed using both Rietveld analysis for whole spectrum (more than 50 reflections) and line profile analysis for the (102), (111) and (221) reflections by mean LIPRAS (Line-Profile Analysis Software^58^) from the calculus of FWHM after a multipeak Pseudo-Voigt peak shape function fitting. Crystallite size and microstrain analysis were calculated using the Caglioti equation^59^ for Rietveld data and the crystallite size for each reflection was calculated using Scherrer equation^60^ and the microstrain broadening (%) is derived by line profile analysis and indicates the microstrain contribution to the total line broadening^61^.

### Thermal gravimetric analysis (TGA)

Thermal gravimetric measurements, differential thermal analysis (DTA) and differential scanning calorimetry (DSC) were carried on using a TA Instruments thermobalance model SDT-Q600 with 0.1 µg of balance sensitivity, located at CAI of Geological Techniques of Complutense University of Madrid (Spain). Powdered samples (10 to 20 mg), held in platinum pans, were heated under a linear gradient from ambient (*ca*. 20 °C) up to 1000 °C; heating rate: 10 °C/min under an N_2_ atmosphere, with flux fixed to 100 ml/min. Two colonies per pH were analysed measuring the weight loss, derivative weight loss and differential heat flow, before the analysis the baseline, DSC precision and temperature was calibrated internally by the laboratory up to five times with different protocols.

## Supplementary information

Supplementary Information

Source data

## Data Availability

The datasets generated during the current study are included in tables in this published article (and its supplementary information files). Moreover, the source data underlying the misorientation barplots of the Fig. 1 are provided as a Source Data file. Materials are housed in the Institute of Paleobiology of Polish Academy of Sciences (PAN).
