# Supplementary material for: Impact of ocean acidification on crystallographic vital effect of the coral skeleton
Source: Nat Commun. 2019 Jul 1;10:2896. doi: 10.1038/s41467-019-10833-6 (PMC6603003; doi:10.1038/s41467-019-10833-6)
Supplement: Supplementary file 1 — Supplementary Information [file 41467_2019_10833_MOESM1_ESM.pdf]

Supplementary Information

Coronado et al., **Impact of ocean acidification on crystallographic vital effect of the coral skeleton**

Supplementary Figures.

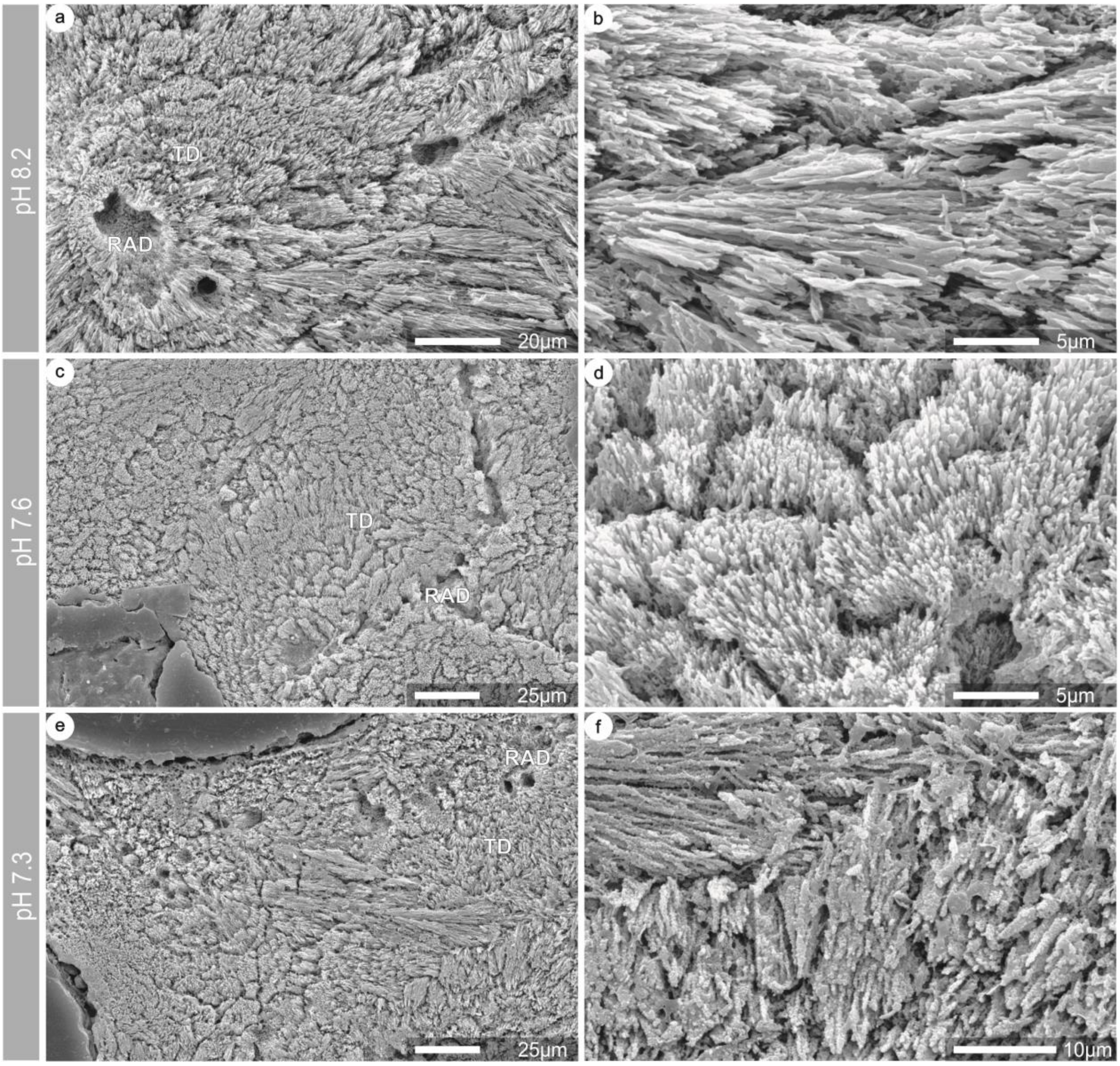

Supplementary Figure 1. **Microstructural features of *Stylophora pistillata* cultured under 3 different pH conditions (8.2, 7.6, 7.3) on etched transverse sections observed under SEM.** (**a**). The crystals of pH 7.3 sample (**f**) have a coarse granular aggregated appearance, with visible intercrystalline organic matrix surrounding them. Crystals of pH 7.6 samples (**d**) are smallest and exhibit an intermediated morphology between usual needle crystals of pH 8.2 sample (**b**) and granular-textured crystals of pH 7.3 sample (**f**).

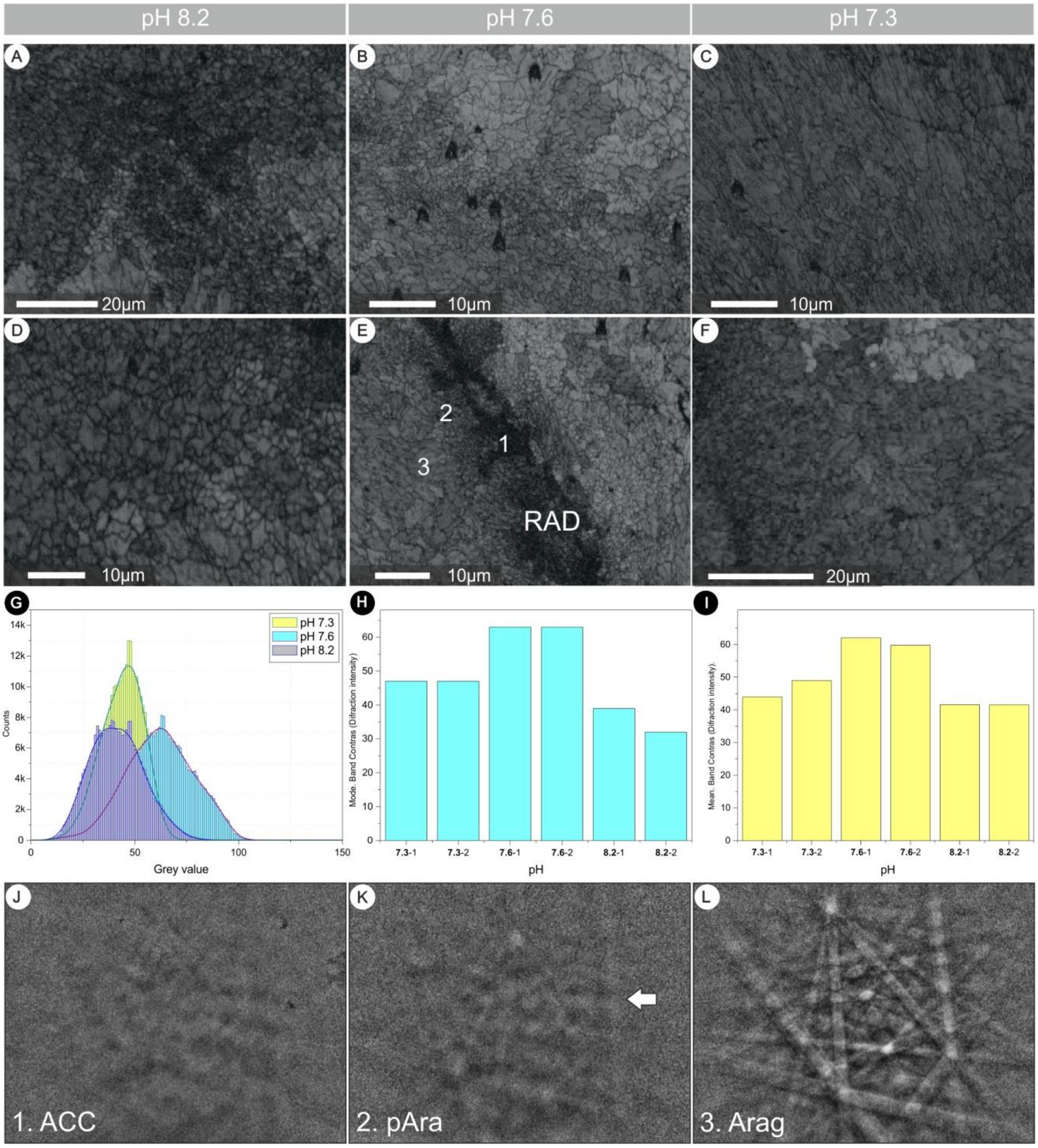

Supplementary Figure 2. **Band contrast (BC) images of skeleton of *Stylophora pistillata* cultured under 3 different pH conditions (8.2, a-d; 7.6, b-e; 7.3, c-f).** The images have been enhanced for publication (30% of brightness and 10% of contrast). RAD: Rapid Accretion Deposits. Numbers in (e) refer to the figures (j-l); **g**) Histograms of pixel intensity of the band contrast images of TD regions, following the methodology proposed by Fitzer et al.[34]. The calculations have been done in not enhanced images. The RAD regions were eliminated of study due to their amorphous nature (DeVol et al.[14]; Mass et al.[15]) and therefore they have low diffraction intensity. **h**-**i**) Bar plots showing the Mode (**h**) and Mean (**i**) of each Band Contrast image, showing that pH 7.6 crystals have high diffraction intensity in comparison with pH 7.3 and 8.2, which are similar, being the distribution of diffraction intensity more constrained in pH 7.3 sample. Note that the bounds between crystals and bundles of fibres present less diffraction intensity at pH 8.2. j-l) Kikuchi patterns of three selected areas of coral skeleton. Amorphous structure of $CaCO_3$ ascribed to possible ACC (1), quasi-amorphous structure ascribed to incipient aragonite or partially disordered protoaragonite (pAra, 2), both in RAD and crystalline aragonite (3) in TD.

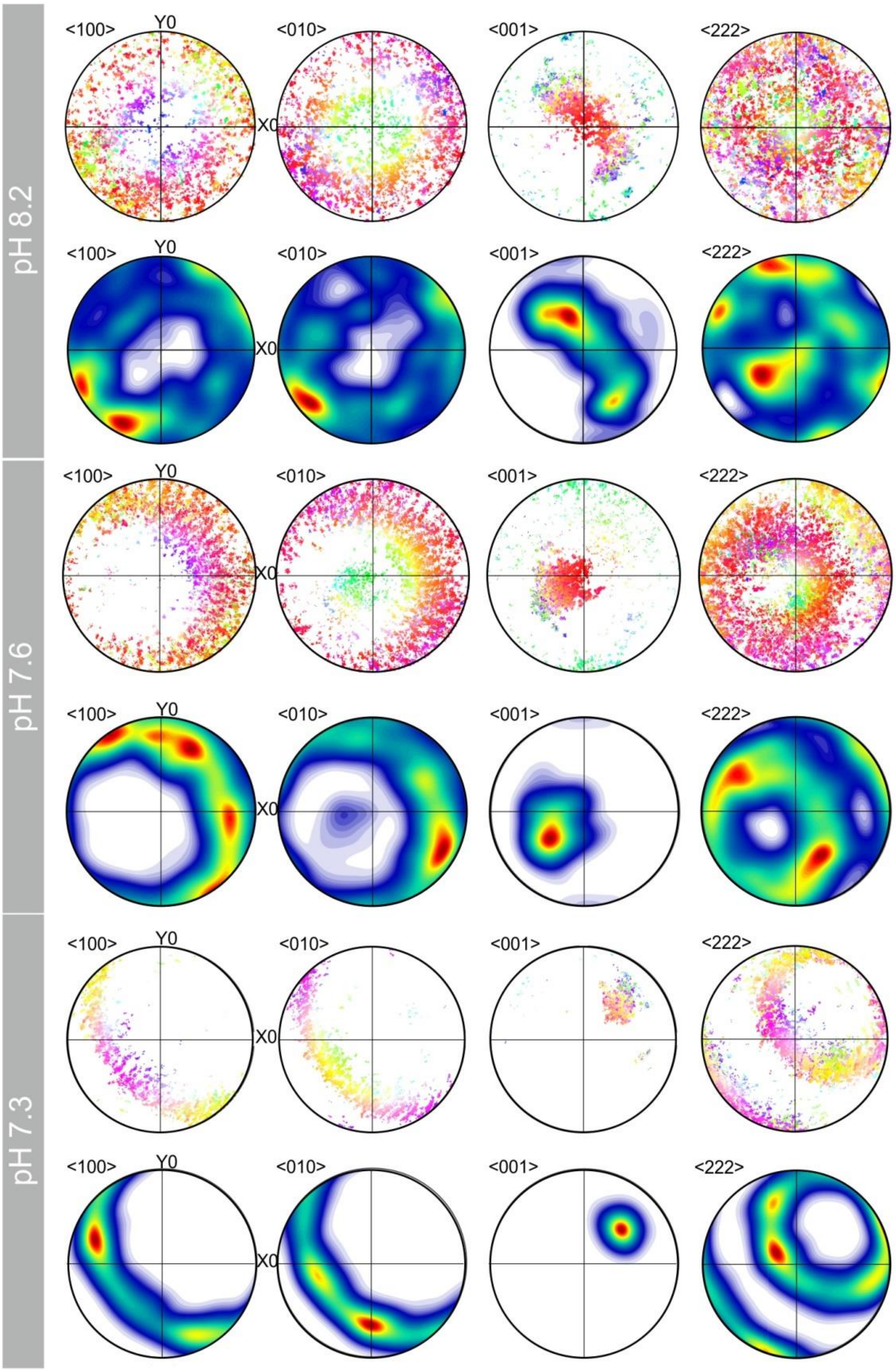

Supplementary Figure 3. **Crystallographic orientation of crystals of skeleton of *Stylophora pistillata* cultured under 3 different pH conditions (8.2, 7.6, 7.3).** Pole figures (in reference direction view (x_0) to the sample surface in a three axes reference system) indicating the crystallographic orientation of aragonite crystals with reference to the planes (100), (010), (001) and (222); and crystallographic key indicating colour coding of crystallographic axes. Orientation density functions, ODF pole figures (in reference direction view (x_0) to the sample surface in a three axes reference system) of the previous pole figures, showing the maxima density orientations.

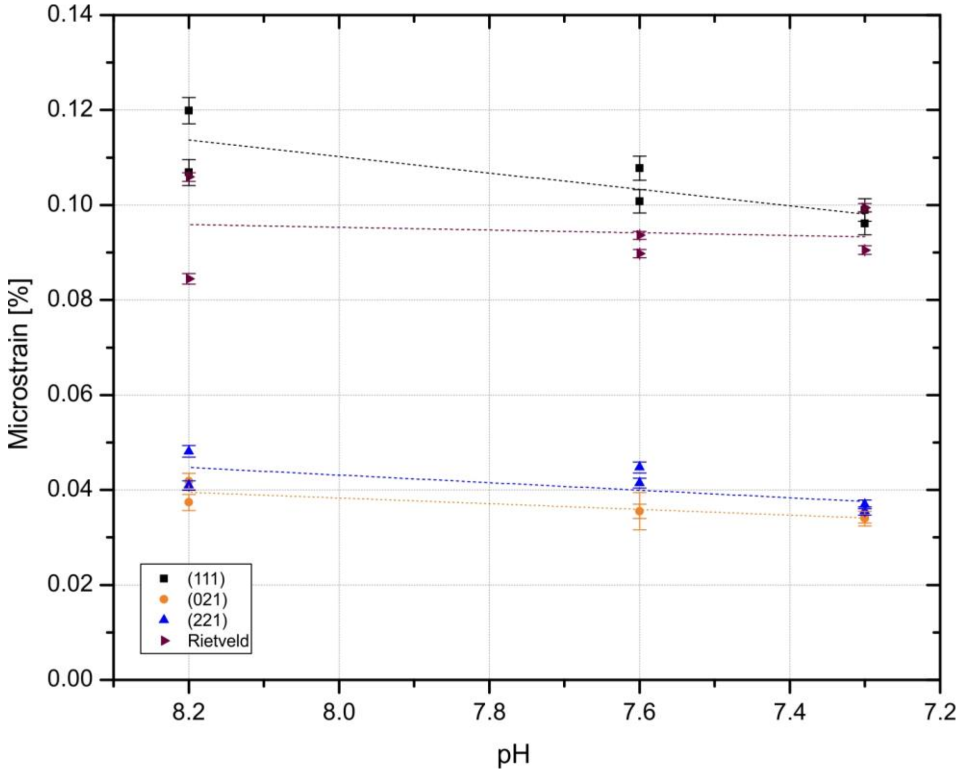

Supplementary Figure 4. **Microstrain fluctuations calculated of skeletons cultured under 3 pH (8.2, 7.6, 7.3).** Note the increasing slope of plane (111) in contrast with the planes (221) and (102). The error bars represent calculated error of macrostrain after refinement of UVW-parameters.

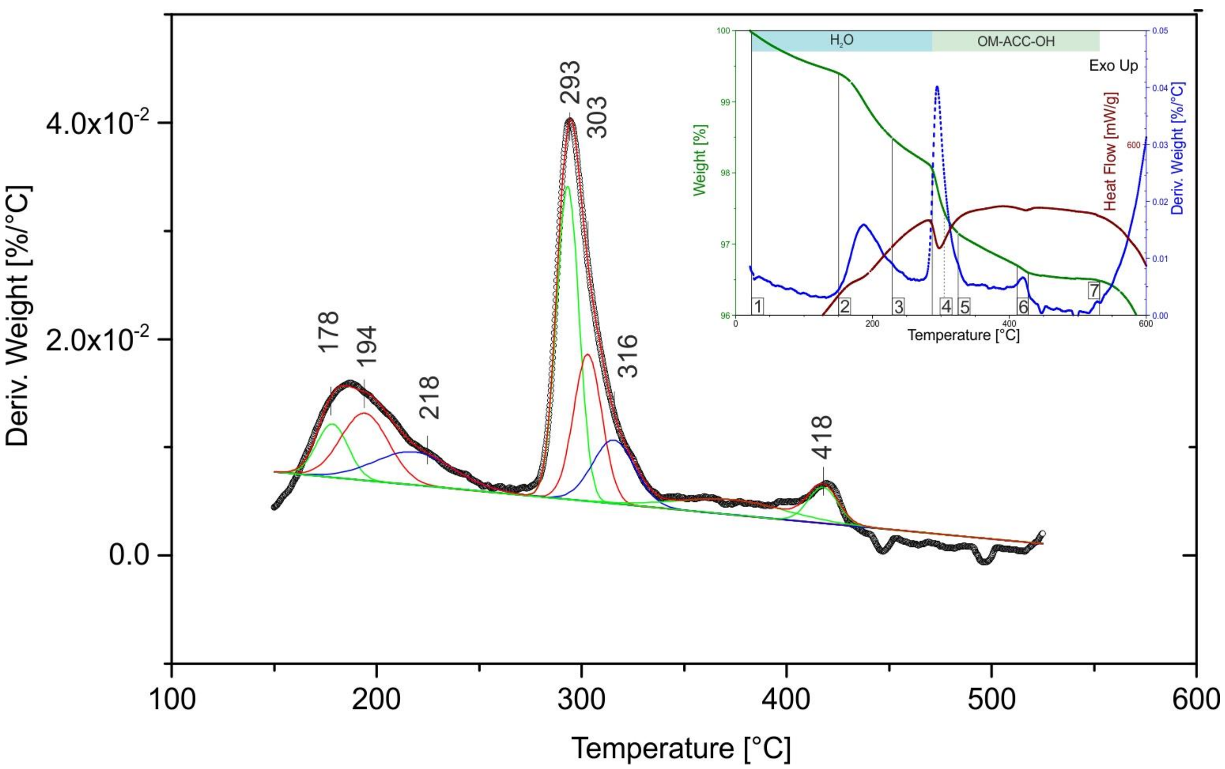

Supplementary Figure 5. **Derivative weight loss profile from sample 8.2-2 pH showing the main exothermal decomposition events deconvoluted. Inset shows the weight loss % (green: 1-7 regions), heat flow (brown, mW/g) and derivative weight loss (blue, %/°C) of the interval 0-600 °C.** Two regions have been differentiated on regard their composition: 1) water (including rigid and fluid molecules *sensu* Schmidt et al., 2014, temperatures from 0-275 °C); 2) A combination of organic matrix (OM), ACC and OH (structural water). Note the temperature of transformation of ACC to a crystalline polymorph (probably calcite) is c.a. 316°C and the total transformation of aragonite to calcite stars c.a. 418°C[44,46] .

Supplementary Tables.

Supplementary Table 1: **Values calculated from Band Contrast images.** S.D.: standard deviation; Min: minimum; Max: maximum.

Sample	Area	Mean	S. D.	Mode	Min	Max	Median
**7.3-1**	298760	43.925	9.877	47	3	77	45
**7.3-2**	219177	48.941	14.231	47	2	112	48
**7.6-1**	298144	62.066	16.161	63	4	117	62
**7.6-2**	145782	59.718	14.842	63	3	114	60
**8.2-1**	255510	41.569	12.685	39	3	91	41
**8.2-2**	188602	41.52	15.558	32	2	255	40

Supplementary Table 2: **Orthorhombic lattice parameters (Å) and unit cell volume (Å^3) determined for aragonite crystals of skeletons cultured under 3 pH (8.2, 7.6, 7.3).** Rietveld refinement factors of structure agreement: R-factors (weighted profile R-factor: Rwp, expected R-factor: Rexp), chi-square or goodness of refinement (χ^2). Macrostrain parameters (Δa/a; Δb/b; Δc/c) calculated from sample of pH 8.2-1.

Sample	χ^2	a	b	c	Δa/a(%)	Δb/b(%)	Δc/c(%)	Rwp	Rexp	Volume
**8.2-1**	2.17	4.969819(19)	7.98070(3)	5.75707(2)	--	--	--	7.38	5.01	228.3407(16)
**8.2-2**	2.47	4.96950(2)	7.98012(3)	5.75684(3)	--	--	--	8.62	5.48	228.3001(17)
**7.6-1**	1.81	4.967223(17)	7.97662(3)	5.75423(2)	-0.0523	-0.0511	-0.0493	7.09	5.26	227.9921(14)
**7.6-2**	2.18	4.967491(17)	7.97693(3)	5.75414(2)	-0.0469	-0.0472	-0.0509	7.54	5.11	228.0099(14)
**7.3-1**	2.94	4.969459(20)	7.98005(3)	5.75660(2)	-0.0072	-0.0081	-0.0082	9.76	5.69	228.2868(17)
**7.3-2**	2.62	4.969363(19)	7.98000(3)	5.75668(2)	-0.0093	-0.0088	-0.0068	9.06	5.6	228.2842(16)

Supplementary Table 3: **Crystallite size and microstrain estimation of aragonite crystals of skeletons cultured under 3 pH (8.2, 7.6, 7.3) using peak broadening equations [61] and Rietveld parameters (Caglioti equation [62],).**

Sample	Reflection	Crystallite size		Microstrain	
		[nm]	**S.D.**	[%]	**S.D.**
		**Peak broadening**			
**8.2-1**	**111**	48.493	1.095	0.1199	0.0027
**8.2-1**	**021**	48.608	1.916	0.0418	0.0017
**8.2-1**	**221**	56.306	1.404	0.0481	0.0012
**8.2-2**	**111**	53.095	1.332	0.1068	0.0027
**8.2-2**	**021**	53.031	2.333	0.0374	0.0017
**8.2-2**	**221**	65.438	1.545	0.0409	0.0009
**7.6-1**	**111**	55.284	1.262	0.1077	0.0025
**7.6-1**	**021**	55.284	2.226	0.0355	0.0039
**7.6-1**	**221**	62.980	1.592	0.0447	0.0011
**7.6-2**	**111**	59.051	1.395	0.1008	0.0024
**7.6-2**	**021**	58.771	2.413	0.0355	0.0015
**7.6-2**	**221**	67.679	1.680	0.0414	0.0010
**7.3-1**	**111**	59.776	1.476	0.0961	0.0024
**7.3-1**	**021**	59.252	2.546	0.0396	0.0015
**7.3-1**	**221**	76.365	1.850	0.0356	0.0008
**7.3-2**	**111**	57.0713	1.351	0.0989	0.0023
**7.3-2**	**021**	57.099	2.364	0.0345	0.0014
**7.3-2**	**221**	71.939	1.759	0.0369	0.0009
		**Rietveld (Caglioti equation)**			
**8.2-1**		39.823	0.165	0.0844	0.0010
**8.2-2**		42.657	0.217	0.1059	0.0009
**7.6-1**		41.309	0.157	0.0897	0.0008
**7.6-2**		45.668	0.206	0.0936	0.0008
**7.3-1**		44.185	0.214	0.0994	0.0008
**7.3-2**		45.933	0.234	0.0905	0.0009

Supplementary Table 4: **Measured weight loss (wt %) during the TG-analysis of skeletons cultured under 3 pH treatments (8.2, 7.6, 7.3).** Table shows the inferred origin of mass loss.

		Samples						
		**8.2-1**	**8.2-2**	**7.6-1**	**7.6-2**	**7.3-1**	**7.3-2**	
	°C			wt %				Area
**Temperatures**	**20-150**	0.663	0.608	0.51	0.523	0.577	0.580	1
	**150-210**	0.677	0.731	0.458	0.438	0.516	0.527	2
	**210-275**	0.531	0.522	0.361	0.377	0.573	0.583	3
	**275-300**	0.578	0.573	0.787	0.74	0.796	0.798	4a
	**300-330**	0.478	0.462	0.416	0.419	0.482	0.486	4b
	**330-411**	0.4613	0.4124	0.389	0.398	0.424	0.438	5
	**411-440**	0.14	0.1234	0.1347	0.1329	0.164	0.179	6
	**440-520**	0.0946	0.0612	0.0679	0.0794	0.086	0.106	7
	**20-275**	1.871	1.861	1.329	1.338	1.666	1.690	H_2O
	**275-520**	1.7519	1.632	1.7946	1.7693	1.952	2.008	OM-OH-ACC
	**Total**	**3.623**	**3.493**	**3.124**	**3.107**	**3.619**	**3.698**	
